# Instagram Video Engagement in Medical Education: Cross-Sectional Study

**DOI:** 10.2196/79124

**Published:** 2026-04-15

**Authors:** Aapeli Kemppainen, Henrik Nuutinen, Virve Koljonen, Petri Kulmala

**Affiliations:** 1 Department of Plastic Surgery, Helsinki University Hospital, University of Helsinki, Fabianinkatu 33, Helsinki, 00100, Finland, 358 50392833; 2 Department of Plastic Surgery, Kuopio University Hospital, University of Eastern Finland, Kuopio, Finland; 3 Education Development and Service Unit, Faculty of Medicine, University of Oulu, Oulu, Finland; 4 Medical Research Center, Oulu University Hospital, Oulu, Finland

**Keywords:** medical education, social media, Instagram, educational videos, learner engagement

## Abstract

**Background:**

Social media (SM) has become an essential tool for delivering formal and informal educational content, including medical education. Instagram-based educational initiatives have shown strong engagement and improved learning outcomes in accounts created for research purposes. However, empirical evidence on engagement with real-life account content remains limited.

**Objective:**

This study aims to identify engaging types of posts, determine the optimal video length for medical education, and provide evidence-based recommendations to medical educators on SM content.

**Methods:**

A cross-sectional study analyzed real-life Instagram medical education account data from Instagram Insights, with a focus on video posts. Insights’ post-related data—reached accounts, views, and watch time—were used. Video posts were categorized based on their type (theoretical, clinical, health promotional, or entertaining) and their implementation (use of animation, human models, or medical models). Video length was stratified into 3 categories: ≤60 seconds, 61-120 seconds, and >120 seconds. The Kruskal-Wallis test was used to determine whether video type, implementation, or length were associated with reach, views, or watch time.

**Results:**

From May 26, 2020, to May 3, 2024, 125 video posts were analyzed. The median of reached accounts was 5317 (IQR 4007-6244) per video. The median (IQR) of views was 6533 (4708-8601) per video, and relative watch time was 19% (15%-30%). Health promotion and awareness videos had the greatest reach (median 7070, IQR 6093-11,340) compared to theory (median 5265, IQR 4476-5829; *P*=.006), clinical skills (median 4597, IQR 2001-5688; *P*=.003), and entertainment videos (median 5605, IQR 3887-6492; *P*=.04). Videos >120 seconds had higher reach (median 5774, IQR 4754-6636) and views (median 7455, IQR 5321-11,031) compared to videos that were 61-120 seconds (median reach 4514, IQR 2551-5615, *P*=.02; views 5987, IQR 3727-6970, *P*=.02). Similarly, videos that were ≤60 seconds had higher reach (median 5484, IQR 4299-6737) and views (median 7189, IQR 5182-10,609) than videos that were 61-120 seconds (median reach 4514, IQR 2551-5615, *P*=.02; views 5987, IQR 3727-6970, *P*=.02). However, videos that were ≤60 seconds had significantly higher relative watch time (median 29%, IQR 20%-40%) compared to videos that were 61-120 seconds (median 17%, IQR 14%-20%; *P*=.003) and videos that were >120 seconds (median 15%, IQR 11%-17%; *P*=.002). The implementation method did not significantly affect video engagement.

**Conclusions:**

Unlike previous studies that have primarily explored the theoretical potential of Instagram or analyzed isolated posts, this study provides a longitudinal real-world analysis of 125 video posts published on an active medical education Instagram account. Our findings demonstrate that shorter video lengths and health promotion content are associated with greater engagement, while diverse implementation types may further support content effectiveness. By examining authentic educational content in SM, this study offers practical, data-driven insights for medical educators seeking to optimize their educational strategies. Future research should explore how different demographic groups engage, thereby enabling more targeted and effective medical education approaches.

## Introduction

Since its launch in 2010, the image-sharing service Instagram has become one of the most popular social networks, with over 1 billion monthly active users [1]. Communication on Instagram relies on short videos, which are suitable for the current fast-paced lifestyle. Social media (SM) platforms have evolved into essential tools for the delivery of both formal and informal educational content [2]. A recent study revealed that health science students spend over 3 hours daily on SM, with 37% of this time dedicated to Instagram [3]. Additionally, a recent survey found that up to 90% of medical and health science students use SM for educational purposes [4].

The use of videos in medical education dates back to the 1960s [5]. Videos are an effective way of communicating knowledge, particularly in medical education [5,6]. Previous studies have demonstrated the effectiveness of videos as educational tools [7,8]. A systematic review found that adding video materials to existing higher education teaching methods significantly improved learning outcomes [9]. For example, compared to standard textbooks, watching a short instructional video before splinting led to faster learning and more successful procedural skills [10]. Video-based education has been shown to enhance knowledge acquisition in surgical training compared to traditional teaching methods [11].

Multimedia learning uses text, narration, pictures, or videos, engaging all senses and minimizing any extra words or pictures, and is suitable even for complex topics [12]. Microlearning involves acquiring knowledge or skills in smaller units [13]. Keeping with the multimedia learning principles and microlearning, 2 comparable eLearning programs on type 2 diabetes treatment were converted into media clips that were 30 to 180 seconds and distributed via SM [14]. The transition from eLearning programs to short SM clips increased participation in the programs by up to 100-fold compared to traditional eLearning programs [14]. An online application was created for emergency medicine guidelines covering 7 topics, for example, chest pain and headache, and was used during shifts by medical students [15]. With the application, they significantly enhanced the frequency and quality of emergency medicine education for undergraduate students in the emergency department [15]. From another standpoint, a family medicine program produced SM videos on evidence-based medicine topics to teach family medicine residents, resulting in significantly improved learner engagement and knowledge acquisition [16]. Although the principle of the cognitive theory of multimedia learning was established already in 2001 [17], it remains applicable to the current medical student Generation Z, as they favor visual and technology-enhanced learning methods [18].

As SM, and especially Instagram, is a widely used tool to share visual material, its educational potential has been recognized [4,19,20]. The use of SM in medical education [21] is a unique combination of open educational resources (OER); social networking; and with regard to videos, multimedia learning. Medical education on SM may use OER and enable just-in-time (JIT) learning to provide resources as needs arise rather than as prescheduled lessons [22]. Aligning with JIT’s on-demand accessibility, SM is well suited for educational purposes via smart devices, either to learn new materials or to revisit what has been learned before. Although OER in medical education is recommended, there are still several obstacles to its widespread use. Such barriers include copyright issues and, especially in clinical contexts, concerns over the use of patient data, photographs, and videos.

Instagram has been instrumental in surgical education, facilitating the sharing of procedural videos and resources to a broad audience while fostering collaboration among students and professionals [23]. Pathologists, in particular, have been active in using Instagram and static images. In previous studies, Instagram accounts developed for histology education demonstrated high usability (98% of respondents), increased learner confidence (95%), and were associated with improved final examination performance [20,24]. Furthermore, an Instagram-based dermatopathology course outperformed a more traditional Canvas platform–based course among a group of 40 dermatology residents [19]. These examples underscore Instagram’s potential to complement traditional learning methods.

SM has been described as promoting social isolation [25,26]. However, SM may also create a sense of communal belonging [27,28]. Interacting with other account followers and content creators by liking, asking, and answering questions in the comment section or through direct messaging is nowadays a new way of expressing sociability. Connectivism, a learning theory, explains learning and teaching in networks using modern technology [29]. It provides insights into using new technology to deliver knowledge, such as diversity, autonomy, openness, and connectivity [30], all of which seem to be fulfilled when using SM in medical education.

Systematic reviews have highlighted the benefits of integrating SM into medical education [31-33]. Despite the increase in research on SM as an educational resource, evidence remains limited regarding the types of content that maximize engagement on platforms like Instagram [34]. Previously, in studies on engagement, accounts have been created solely for study purposes [16,19,20,24]. There is a paucity of research on the engagement of educational SM posts in real-life medical education accounts.

Therefore, we analyzed engagement metrics for medical education posts from a real-world educational SM account using a cross-sectional design. We also aimed to identify the most engaging types of videos by analyzing several features, thereby providing real-life recommendations for effective medical education content on SM.

## Methods

### Ethical Considerations

The study protocol was reviewed and approved by the University of Helsinki Ethical Review Board in Humanities and Social and Behavioral Sciences (statement 65/2024). The study was conducted as a secondary analysis of aggregated SM analytics data and did not involve direct interaction with human participants or the collection of identifiable personal data.

The data were obtained from Instagram Insights, which provides anonymized and aggregated post-level metrics, including reach, views, comments, and average watch time, as well as aggregated follower demographics, such as age group and gender distribution. Instagram Insight data have been used for scientific purposes in previous studies [20,35]. No individual-level data were accessible to the researchers, and individual users could not be identified from the dataset. Since the study analyzed, anonymized, and aggregated data without participant recruitment or intervention, individual informed consent was not collected. No compensation was provided.

Some video posts analyzed in this study included individuals appearing in publicly available content on the account. All individuals who appeared in the videos had provided permission for the publication of the content on the SM platform. No additional identifying information is included in the manuscript or supplementary materials, and no individuals can be identified from the reported data.

### Instagram Account

*Plastiikkaope* (in English, *Plasticsteacher*) is a nonprofit Instagram account created and maintained by one of the authors (VK) to share surgical educational material through short videos and pictures. Targeting medical students, its content focuses on surgery. Posts adhere to multimedia learning principles, with written explanations, narratives, and key points included either in the videos or in the comments section. As a public account, it is accessible to everyone aged 16 years and older. The *Plasticsteacher* account was created in May 2020, with the first post published on May 26, 2020.

### Data

Data were collected on July 9, 2024, by 1 author (AK) using Instagram’s analytics tool, Instagram Insights. All posts published before data collection were included in the initial analysis. The first post was published on May 26, 2020, and the latest on May 3, 2024. The sampling of the posts was not conducted.

To study the popularity and further engagement of the posts, we collected reach, views, and watch time as post-related parameters from Insights. Reach refers to the number of unique accounts that viewed a post at least once after its release, while views indicate the number of times a video starts playing. Starting from September 27, 2023, updates in the Insights software enabled the separate analysis of initial plays and replays. Insights also provided watch time metrics such as total watch time and average watch time from September 2022 onwards. A relative watch time was calculated by dividing the average watch time by the length of a video. Not all post-related parameters were available for each post, not only because of updates on the Insights analytics application but also for technical reasons. For carousel posts, which contain multiple videos or pictures in 1 post, Insights provided reach, but views or watch time metrics were not provided. From September 2023 onwards, Instagram Insights divided views into initial plays and replays.

In addition to post-related parameters, we collected the account’s follower metrics, including the followers’ age range, gender, and geographic locations. The follower data were visible for only the previous 90 days in Insights, meaning that the follower data represented the demographics of followers between April 10, 2024, and July 9, 2024, limiting the analysis of follower development.

Collaborative posts with other nonprofit accounts and posts with pictures only were excluded from the analysis. Included posts were stratified into educational and noneducational (ie, entertainment) groups and further divided into subgroups by authors. Educational posts were further divided into three content categories: (1) theory, (2) clinical skills, and (3) health promotion and awareness. Educational posts were also classified into three video implementation types: (1) animated videos, (2) videos using medical models (phantoms), and (3) videos using human participants. All individuals appearing in the videos were healthy volunteers who gave their consent to be featured. Typically, these individuals included fellow doctors, nurses, and relatives of author VK. Videos were categorized into 3 groups based on their length: ≤60 seconds, 61-120 seconds, and >120 seconds.

The analysis focused on video posts. First, we examined the distribution of different videos over the period investigated. On the basis of these findings, videos were selected for a second analysis, in which statistical differences were tested between video categories, implementation types, and length groups under 3 parameters: reached accounts, video views, and relative watch time.

### Statistical Analysis

Statistical analysis was conducted using IBM SPSS Statistics version 29.0.2.0. Data were tested for skewed distribution by visual inspection of histograms. To assess statistical significance between subgroups based on length, video content, and video implementation, the independent-samples Kruskal-Wallis test was applied, followed by the Dunn pairwise comparisons test. Benjamini-Hochberg post-hoc corrections were applied in pairwise comparisons since the nature of the study was exploratory, and multiple subgroups were tested. Post hoc corrections were calculated in Microsoft Excel version 16.103.3 based on pairwise *P* values. For *P* values reported by SPSS as *P*<.001, a value of .001 was used for multiple-comparison correction, resulting in conservative estimates. A *P* value of ≤.05 was considered statistically significant. For missing data, the Little missing completely at random (MCAR) test was performed.

## Results

### Overview

**Figure 1.**
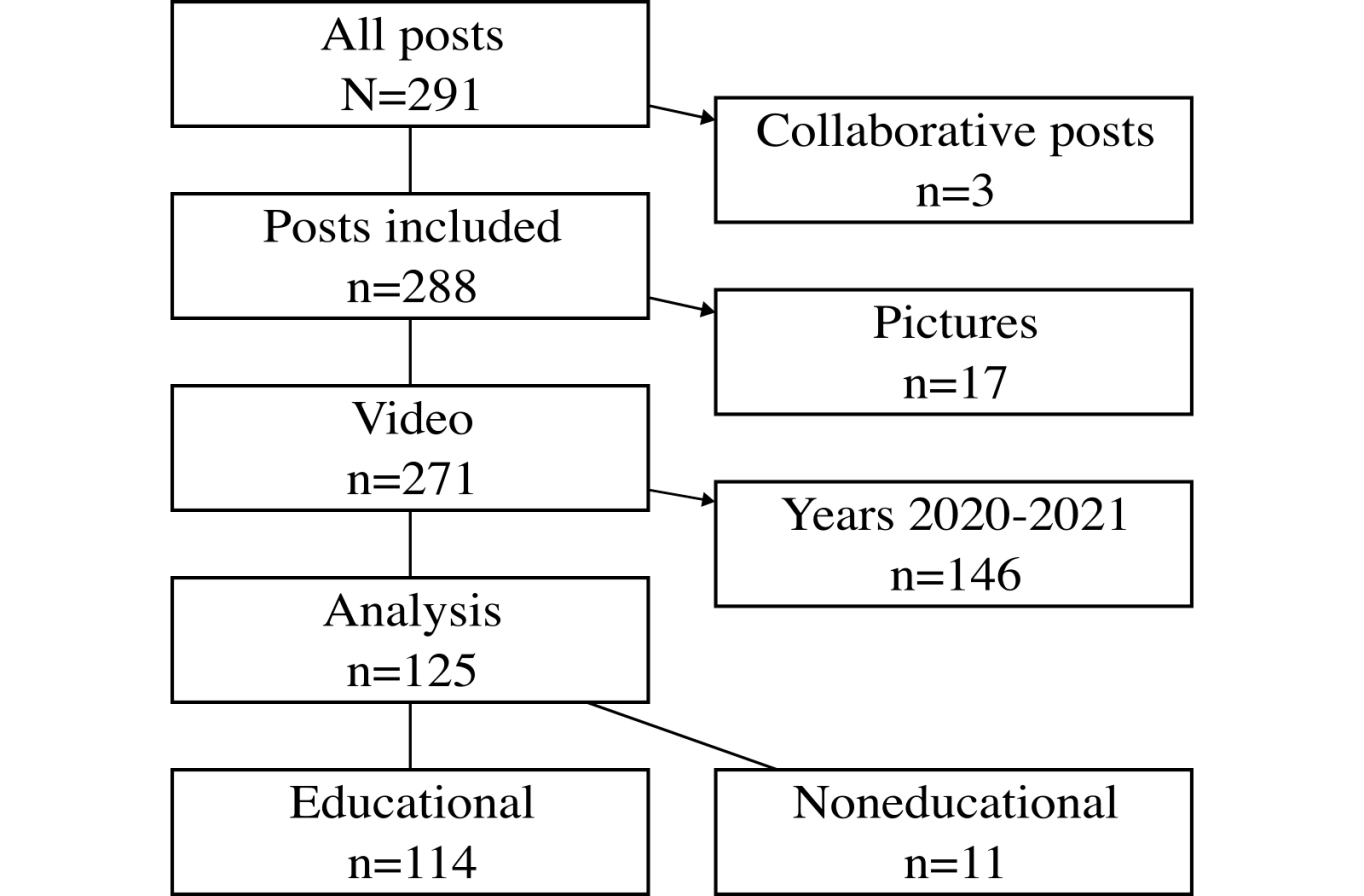
Flowchart of study analysis. Collaborative posts, pictures, and posts during 2020‐2021 were excluded from the final analysis. A more detailed description of the last phase of exclusion is shown in Multimedia Appendix 1.

**Figure 2.**
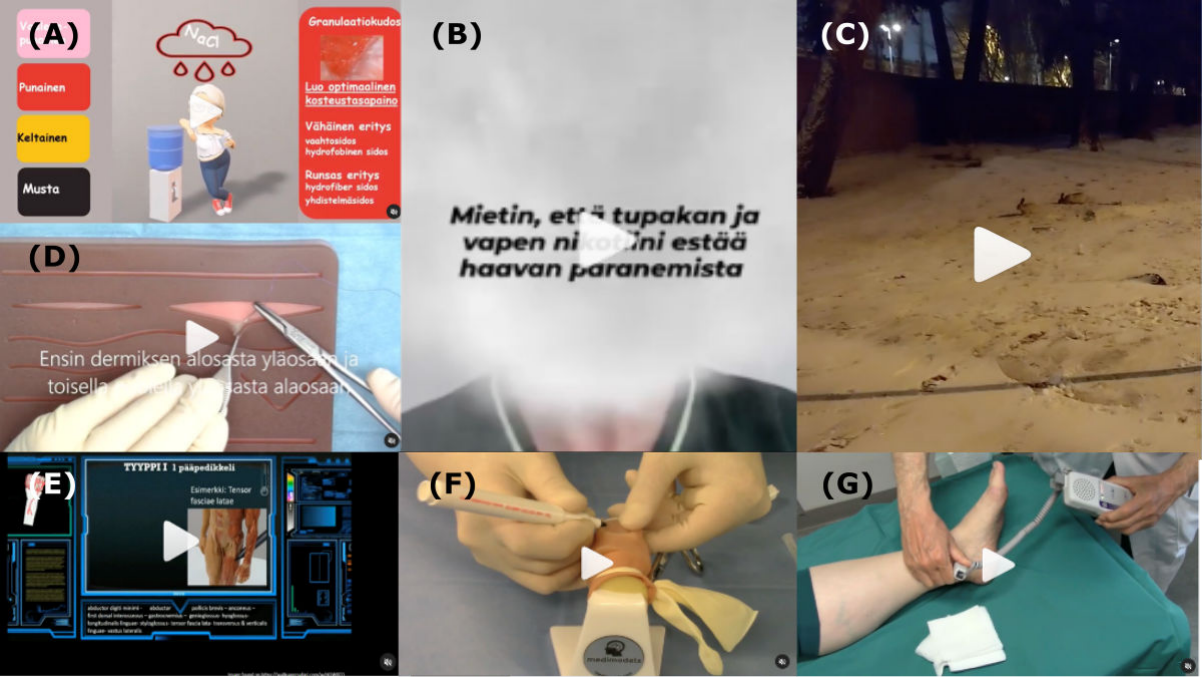
Examples of different video categories and implementation types. (A) Theory wound bed classification using animation, (B) health promotion and awareness using a filter on how nicotine in tobacco and vape inhibits wound healing, (C) an entertainment video about rabbits from a nearby hospital, (D) clinical skills showing how to make an inverted dermal suture, (E) animation presenting classification of flaps, (F) using a medical model to demonstrate an ingrown toenail operation, and (G) using a healthy volunteer (author VK) to demonstrate examining pulse status in the lower extremity.

From May 26, 2020, to May 3, 2024, 291 posts were identified. Of these, 125 posts were included in the final analysis (Figure 1). Videos considered as health promotion and awareness and lasting ≤60 seconds were not published until 2022. The median values were lower for reached accounts (551, IQR 433-763 vs 5317, IQR 4010-6205) and views (1695, IQR 1415-2128 vs 6533, IQR 4756-8573) for videos posted between 2020 and 2021 compared to videos posted between 2022 and 2024. On the basis of these findings, the videos posted between 2020 and 2021 were excluded to avoid underestimating the median reach and views of other subgroups, including theory, clinical skills, entertainment, and videos lasting 61‐120 and >120 seconds. The MCAR test was performed for all videos included in the final analysis (N=125), and missing data were not replaced. The percentage of missing data was low for reached accounts (0%) and views (2.4%), while the percentage was moderate for watch time (24%) and high for initial or replay views (76.8%), which is explained by developmental updates on Instagram Insights. Figure 2 illustrates categories under video content and video implementation type. A detailed description of the exclusion criteria for 2020‐2021 posts and MCAR testing is provided in Multimedia Appendix 1.

### Follower Metrics

As of June 9, 2024, the account had 7538 followers, 83% of whom were females. Most followers were from Finland (98%). The largest age group was 25‐34 years (44%), followed by 35‐44 years (26%) and 45‐54 years (13%). Among female followers, 41% were aged 25‐34 years, while 60% of male followers were in this age group. Follower metrics were available in Instagram Insights for 90 days prior to the data collection date, so the development of follower demographics could not be analyzed.

### Posts

**Figure 3.**
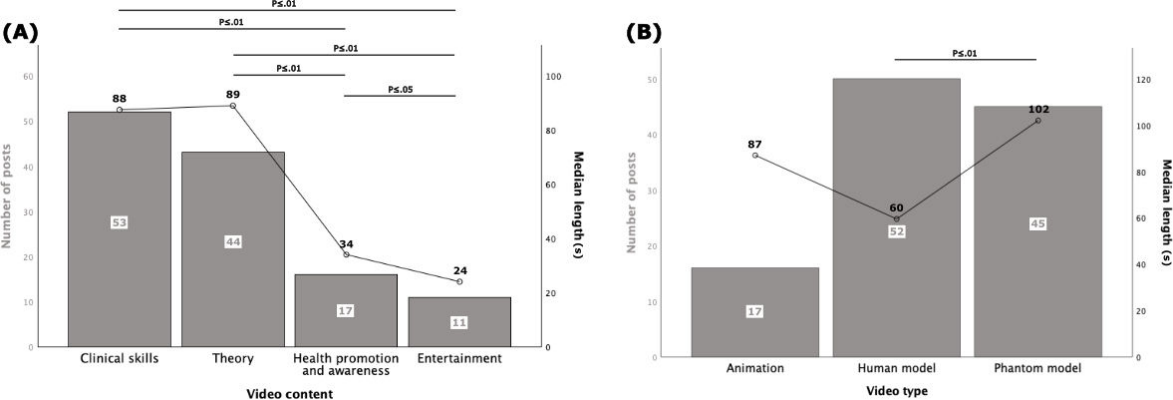
Video classification based on video content, implementation type, and comparisons between video lengths of the categories. (A) Columns refer to the number of posts under different subgroups. Of the included 125 videos, 42% (n=53) were clinical skills videos, 35% (n=44) were theory videos, 14% (n=17) were health promotion videos, and 9% (n=11) were entertainment videos. Data points refer to the median video length and are shown with a connecting line for visualization purposes only. Median (IQR) video lengths were 89 (63-110) seconds for theory videos (n=43), 88 (60-138) seconds for clinical skills videos (n=52), 34 (23-53) seconds for health promotion and awareness videos (n=16), and 24 (16-29) seconds for entertainment videos (n=11). Theory and clinical skills videos were significantly longer than health promotion and awareness videos and entertainment videos pairwise. Health promotion and awareness videos were significantly longer than entertainment videos. There was no statistically significant pairwise difference between theory and clinical skills videos. (B) Among educational videos (n=114), human models were used in 46% (n=52), phantom models in 39% (n=45), and animations in 15% (n=17). Median (IQR) video lengths were 102 (72-185) seconds for phantom model videos (n=45), 87 (54-106) seconds for animated videos (n=16), and 60 (35-91) seconds for human model videos (n=50). Phantom model videos were significantly longer than human model videos. There was no statistically significant difference between other groups pairwise. Exact *P* values are listed in Multimedia Appendix 1.

Of the 125 videos included, most (n=53, 42%) were clinical skills videos (Figure 3A). Among educational videos (n=114, 91%), human models were used most frequently (n=52, 46%; Figure 3B). Regarding video length, 40% (n=51) were ≤60 seconds, 41% (n=51) were 61‐120 seconds, and 19% (n=23) were >120 seconds.

The median (IQR) video length of the analyzed videos (N=125) was 72 (42-109) seconds. The longest median (IQR) length was recorded as 89 (63-110) seconds for theory videos (n=43). Theory and clinical skills videos were significantly longer than health promotion and awareness videos and entertainment videos in pairwise comparisons (theory vs health promotion and awareness, *P*=.006; theory vs entertainment, *P*=.003; clinical skills vs health promotion and awareness, *P*=.002; clinical skills vs entertainment, *P*=.002; Figure 3A). Health promotion and awareness videos were longer than entertainment videos (*P*=.04). There was no statistically significant pairwise difference between clinical skills and theory videos.

By video implementation types, the longest median (IQR) video lengths were 102 (72-185) seconds for phantom model videos (n=45). Phantom model videos were significantly longer than human model videos (*P*=.003; Figure 3B). There was no statistically significant difference between other groups in pairwise comparisons.

### Relative Watch Time

**Figure 4.**
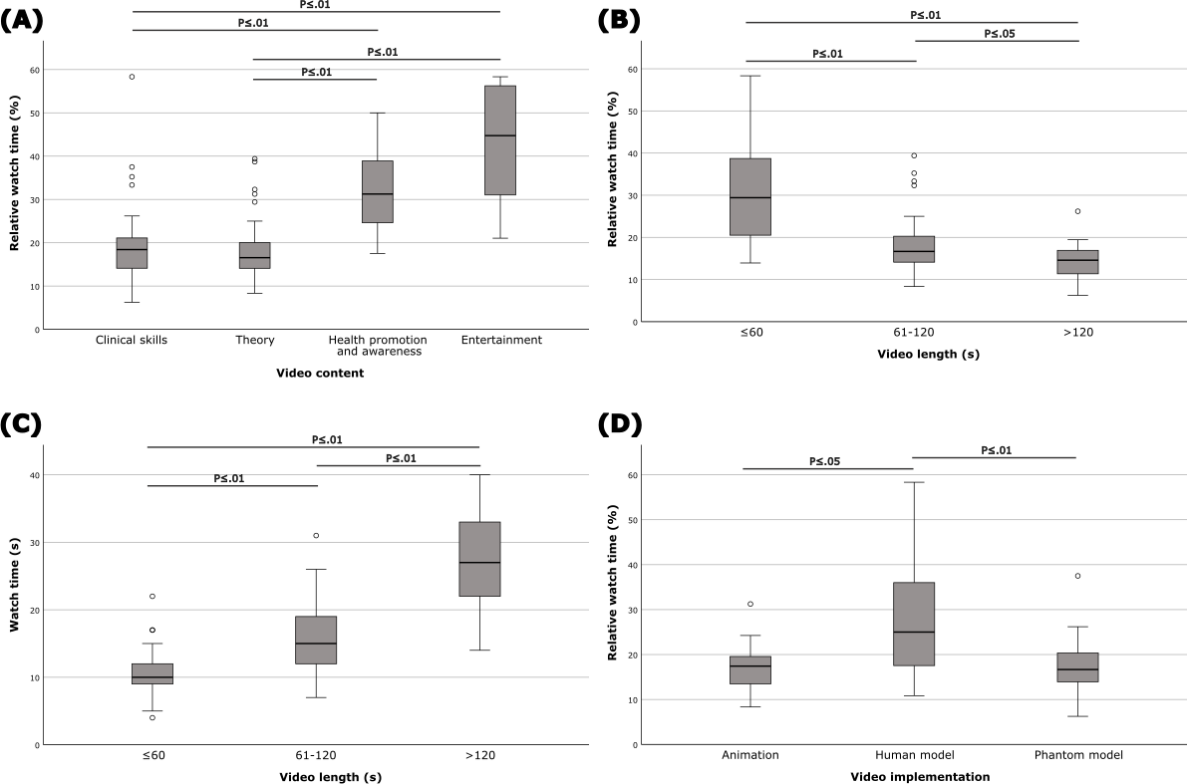
Statistical analysis of relative watch time for different video content categories, video length groups, and video implementation types. (A) Health promotion and awareness videos and entertainment videos were significantly more watched than theory and clinical skills videos when compared pairwise, but there was no statistically significant difference between health promotion and awareness videos and entertainment or between theory and clinical skills videos. (B) The shortest length videos (≤60 s) were watched significantly more compared to other video length groups, followed by videos that were 61‐120 seconds and videos that were >120 seconds; the difference was statistically significant between all groups when compared pairwise. (C) Videos longer than 120 seconds had the significantly longest watch time, followed by videos that were 61‐120 seconds and videos ≤60 seconds; the difference was statistically significant between all groups when compared pairwise. (D) Human model videos had significantly higher relative watch time compared to animated and phantom model videos. Exact *P* values are listed in Multimedia Appendix 1.

The median relative watch time for the 95 (76%) videos with available watch time data was 19% (IQR 15%-30%). Insights provided total watch time and average watch time from September 2022 onwards; therefore, data were not available for posts published earlier in 2022 (30 video posts). The highest median (IQR) relative watch time was 45% (26%-56%) for entertainment videos (n=9). Entertainment and health promotion and awareness videos were watched significantly further relative to their length compared to theory and clinical skills videos (entertainment vs theory, *P*=.003; entertainment vs clinical skills, *P*=.002; health promotion and awareness vs theory, *P*=.006; health promotion and awareness vs clinical skills, *P*=.002; Figure 4A).

When analyzed by video length, the highest median (IQR) relative watch time was 29% (20%‐40%) for videos 60 seconds or less (n=41). The shortest videos (≤60 seconds) were watched significantly more compared to other length groups (≤60 seconds vs 61-120 seconds, *P*=.003; ≤60 seconds vs >120 seconds, *P*=.002), followed by videos that were 61 to 120 seconds that were watched significantly more than videos that were more than 120 seconds (*P*=.04; Figure 4B). To complete the previous finding, an average watch time of the video length groups was analyzed. From the analysis, videos that were more than 120 seconds had statistically significantly the longest watch time, and videos that were 60 seconds or less had the shortest watch time (>120 seconds vs 61-120 seconds, *P*=.001; >120 seconds vs ≤60 seconds, *P*=.002; 61-120 seconds vs ≤60 seconds, *P*=.003; Figure 4C), meaning the longer the video, the longer the absolute watch time on average.

By video implementation type, the highest median (IQR) relative watch time was 25% (17%‐36%) for human model videos (n=37). Human model videos had statistically significantly higher relative watch time compared to other video types (human model vs phantom model, *P*=.003; human model vs animation, *P*=.01; Figure 4D).

### Reach

**Figure 5.**
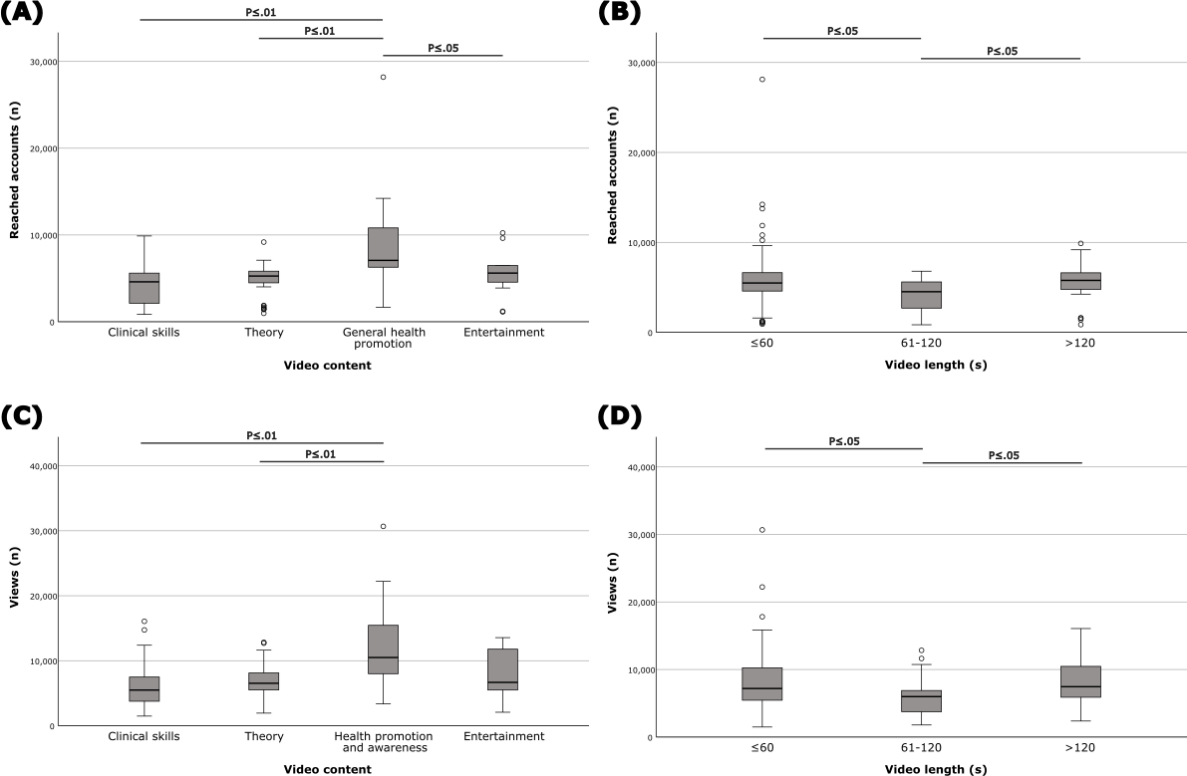
Statistical analysis of reached accounts and views for different video content categories and video length groups. (A) Health promotion and awareness videos reached more accounts compared to all other categories, with a statistically significant difference. There were no significant differences between the other categories. (B) Videos ≤60 seconds and >120 seconds reached significantly more accounts compared to videos 61‐120 seconds in length, but there was no significant difference between videos that were ≤60 seconds and videos that were >120 seconds. (C) Health promotion and awareness videos had significantly more views compared to theory and clinical skills videos. There were no statistically significant differences between the other categories. (D) Videos ≤60 seconds and >120 seconds were significantly more viewed compared to videos 61‐120 seconds in length, but there were no significant differences between videos that were ≤60 seconds and videos that were 120 seconds. Exact *P* values are listed in Multimedia Appendix 1.

The reach demonstrates the number of unique users who saw the post, highlighting the audience size. The median (IQR) reach was 5317 (4007-6244) unique accounts per video for 125 analyzed videos. The most popular videos were health promotion and awareness videos (n=17), with a median (IQR) of 7070 (6093-11,340) reached accounts. Health promotion and awareness videos had a significantly higher reach compared to all other categories, while no statistically significant differences were observed between theory, clinical skills, and entertainment videos (health promotion and awareness vs clinical skills, *P*=.003; health promotion and awareness vs theory, *P*=.006; health promotion and awareness vs entertainment, *P*=.04; Figure 5A).

When analyzed by video length, the most popular were videos that were more than 120 seconds (n=23) with a median (IQR) of 5774 (4754-6636) reached accounts. Videos with the length of ≤60 seconds and >120 seconds had significantly higher reach compared to videos 61‐120 seconds in length, but no statistically significant differences were found between the longest and shortest videos (≤60 seconds vs 61-120 seconds, *P*=.02; >120 seconds vs 61-120 seconds, *P*=.02; Figure 5B). Comparing video implementation types (animation, human, and phantom), there was no statistically significant difference.

### Views

The data were available for 122 (98%) videos. The median (IQR) views were 6533 (4708-8601) per video. The highest median (IQR) views were 10,499 (7792-15,650) for health promotion and awareness videos (n=16). Health promotion and awareness videos had significantly more views compared to theory and clinical skills videos (health promotion and awareness vs theory, *P*=.006; health promotion and awareness vs clinical skills, *P*=.003). There were no significant differences between other groups in pairwise comparisons (Figure 5C).

When analyzed by video length, the highest median (IQR) views were 7455 (5321-11,031) for videos that were more than 120 seconds (n=23). The longest videos (>120 seconds) and the shortest (≤60 seconds) reached more views compared to videos that were 61 to 120 seconds, but no statistically significant differences were observed between the longest and shortest videos (≤60 seconds vs 61-120 seconds, *P*=.02; >120 seconds vs 61-120 seconds, *P*=.02; Figure 5D). Comparing video implementation types (animation, human, phantom), no statistically significant differences were found.

From September 2023 onwards, Instagram Insights divided views into initial plays and replays. We analyzed the replays for videos published after September 2023 (n=29). The median (IQR) percentage of replays for the videos was 36% (35%-38%) of all views. When comparing video content, length, or implementation type, no statistically significant differences were found.

## Discussion

In this study, we analyzed the content of a real-life SM account focused on medical education to determine which types of content were most well received by followers. To the best of our knowledge, no previous studies have performed a longitudinal real-life content analysis of a similar Instagram account [23,31-33]. By focusing exclusively on videos, this study provides new insights into their educational value. We specifically studied videos shared on Instagram, which is an applicable platform for short instructional educational videos applying the principles of multimedia learning and microlearning [4,17,36].

Health promotion and awareness posts were particularly successful in reaching audiences, as they reached most accounts (health promotion and awareness vs clinical skills, *P*=.003; health promotion and awareness vs theory, *P*=.006; health promotion and awareness vs entertainment, *P*=.04). As the World Health Organization (WHO) recognizes, SM platforms provide unique opportunities for promoting health awareness and prevention [37]. Video-based educational interventions have previously been used to promote health awareness and prevention by professionals, researchers, and a variety of organizations [38,39]. For example, video interventions resulted in more targeted short-term health behaviors such as sunscreen adherence and breast self-examination, although the data are not uniform [39]. Our data support previous findings, as health promotional videos reached the widest audience. These videos could be integrated into broader organizational educational campaigns. *Plasticsteacher*’s health promotion and awareness videos were usually inspired by and produced to match SM trends, which could at least partially explain their popularity. There might be an opportunity to both educate students and spread medical knowledge by combining medical education with SM trends [21,29].

The median length of *Plasticsteacher* videos was 72 seconds, which is longer than the previously found average length of 33 seconds for medical posts on SM [40]. Health promotion and awareness videos, as well as entertainment videos, were significantly shorter than theory and clinical skills videos (health promotion and awareness vs theory, *P*=.006; health promotion and awareness vs clinical skills, *P*=.002; entertainment vs theory, *P*=.003; entertainment vs clinical skills, *P*=.002). We aimed to seek the optimal length for educational videos by assessing views, reaches, and relative watch time. Both the videos that were 60 seconds or less or more than 120 seconds were evenly reached and viewed, yet still statistically significantly better than videos that were 61 to 120 seconds (≤60 seconds vs 61-120 seconds, *P*=.02; >120 seconds vs 61-120 seconds, *P*=.02). However, the shorter videos (≤60 seconds) were watched significantly further when comparing the relative watch time (≤60 seconds vs 61-120 seconds, *P*=.003; ≤60 seconds vs >120 seconds, *P*=.002). Hence, the shortest videos (≤60 seconds) were most effective to deliver information, but longer videos (>120 seconds) were equally popular. Although previous qualitative and experimental studies have demonstrated that shorter videos were more engaging and university students were more inclined to rewatch videos [41], our study indicates that a variety of video lengths may be suitable for medical education on SM. The shortest video length in our study was 9 seconds, and the longest was 6 minutes and 16 seconds. Microlearning, which involves acquiring information in small units, serves as an emerging educational technique in medical education [36,42]. Depending on the literature, a microlearning lesson can be up to 5 minutes long or even up to 15 minutes in length, although students’ focus tends to decrease after 9 minutes [36]. On the basis of our findings, short videos, adhering to the principles of microlearning, could be used to help students prepare for exams, such as reviewing the key steps of surgical procedures.

Although health promotion and awareness videos were the most popular, clinical skills, theory, and entertainment videos performed equally well, with no significant differences in reach or views between these categories. *Plasticsteacher* is targeting medical students on surgical topics, with views having an IQR of 4708 to 8601, which may indicate that videos of all categories are seen as meaningful. Interestingly, the video implementation type—whether using human models, phantom models, or animations—did not significantly impact video popularity, suggesting that producing a variety of video types may be beneficial when planning educational content. Different visualization methods, such as animations for explaining surgical techniques or human models for realistic demonstrations, can complement each other and enrich the learning experience [17]. However, the data on whether video implementation influences popularity or educational outcomes remain limited in the current literature [31-33]. For reference, in SM marketing, pictures with human faces and filters received more reactions compared to pictures without human faces or those that were unfiltered [43].

*Plasticsteacher* publishes posts in Finnish, which we see as an explanation for 98% of followers being from Finland. Eighty-three percent of the followers were females, and the largest age group was 25 to 34 years, with 44% representation. The primary target population for *Plasticsteacher* is medical students, with a secondary focus on other health care professionals. A survey was conducted among 4th to 6th year Finnish medical students, with a median age of 25 years and an age range of 21-42 years [44]. Similar results have been surveyed in Austria, where the mean age of medical students was 24.7 years, with an age range of 21‐32 years [45]. Females have been over-represented in surveys of medical students, with 51% to 58% representation [44,45], but globally, as in Finland, professional care work is a female-dominated sector [46]. Based on the findings, we see that demographic data from Insights are in line with the followers *Plasticsteacher* is aiming to reach.

Videos on Instagram are easily accessible and provide a suitable platform for educational videos when needed [34]. The mobile app as a platform enables viewers to use videos for JIT learning to obtain information when needed [13]. It has been shown that a JIT learning–based mobile app enhanced the quality of medical education during on-call shifts in the emergency department [15]. Similarly, a short instructional video on splinting yielded better outcomes in terms of learning and skills [10]. Our analysis underlines the potential of Instagram videos as an educational tool, as 36% of views were replays, showing that, on average, more than half of the videos were watched again after the initial view.

Despite these strengths, this study has some limitations. The basis for parameter selection was that reached accounts and views could be seen as objective parameters for general popularity. Further, relative watch time may have indicated whether the video was considered beneficial by the viewer. The wide variability observed in reach, views, and watch times across categories may reflect the influence of Instagram’s algorithms, which can cause posts to go viral unpredictably [43]. As in most retrospective studies, the data were not comprehensive and caused limitations for result interpretations. The development of Instagram Insights analytics during the study period also meant that some metrics were not consistently available for older posts. For example, Insights provided total watch time and average watch time beginning September 27, 2022. Nevertheless, these limitations were systematic, considering all types of posts similarly and did not compromise the overall findings.

Additionally, follower metrics including followers’ age range, gender, and geographic data were visible for only the previous 90 days. A recent questionnaire study evaluated the engagement and impact of an educational veterinary Instagram account [47]. It demonstrated a high engagement rate and a positive impact on knowledge and practical skills among veterinary students [47]. Similar studies in the medical field would enable a more comprehensive analysis of followers, which was prevented by the Instagram Insight data limitation.

In conclusion, this study provides a unique analysis of video-based real-life educational content on Instagram. The study emphasizes the opportunity to produce high-quality medical content for the public without using actual patient data. The role of social media as a platform for medical education has been increasing [31-34], yet there remains a need to develop educational material that is evidence-based [2]. There is a clear gap in analytical research on this topic, in which this study stands out as a pioneering effort. Our findings highlight the effectiveness of short video length and health promotional content in engaging viewers, while also demonstrating the value of diverse video implementation types. We observed that demonstrative video content is well received by audiences. Health promotion content can serve as inspiration for medical professionals, and based on its founded effectiveness, it may be beneficial to incorporate such content into larger educational campaigns on SM. Short videos can be used in diverse ways on SM, such as summarizing key points on theoretical topics or reviewing key steps of procedures. Future studies should explore the impact of Instagram’s algorithms on variations in video popularity and further examine how different demographic groups engage with educational content. Such studies would support the development of a more targeted and effective approach to medical education content creation.

## Supplementary material

10.2196/79124 Multimedia Appendix 1 Supplementary methodological analyses: exclusion of 2020-2021 posts, missing completely at random test, and adjusted *P* values (Benjamini-Hochberg corrections).

## Abbreviations

JIT: just-in-time
MCAR: missing completely at random
OER: open educational resources
SM: social media
WHO: World Health Organization
