# Supplementary material for: Instagram Video Engagement in Medical Education: Cross-Sectional Study
Source: JMIR Med Educ. 2026 Apr 15;12:e79124. doi: 10.2196/79124 (PMC13082447; doi:10.2196/79124)
Supplement: Multimedia Appendix 1 [file mededu-v12-e79124-s001.docx]

**Supplementary material**


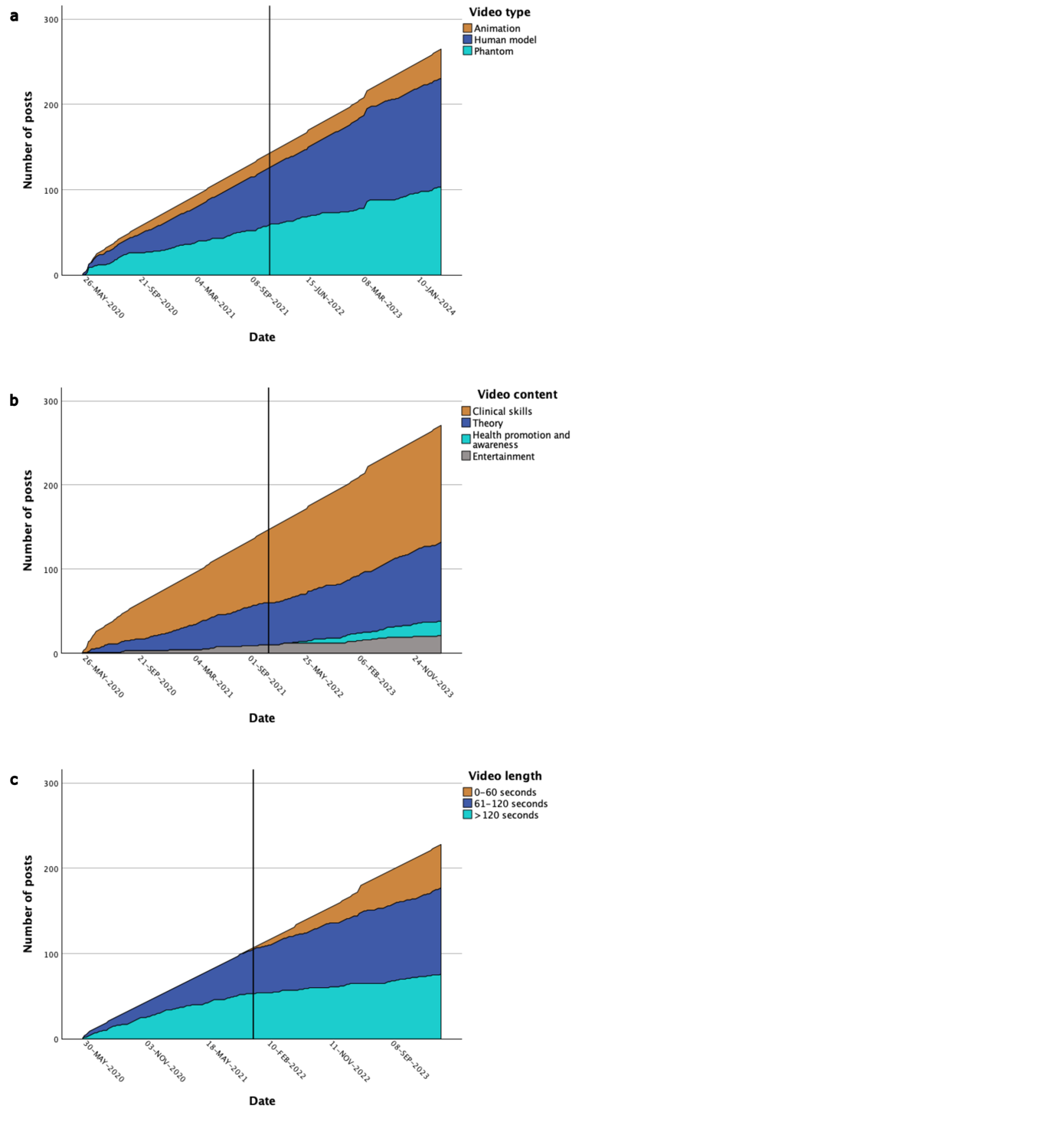


**Figure S1 | Exclusion of 2020 – 2021 posts.** Videos posted between 2022 and 2024 (n = 125) were included in the final analysis. Vertical line in the figures illustrates the point from which forward videos were included into the analysis. **a** Videos under different video implementation types as human model, phantom model and animation were posted consistently from 2020 onward. **b** The first health promotion and awareness video was posted on 24^th^ March 2022, and since then, posts were published in all categories: theory, clinical skills, health promotion and awareness, and entertainment. **c** Videos in all length groups began appearing after the first video in the shortest group (≤60 seconds) was posted on 28^th^ October 2021.

Since health promotion and awareness and the shortest ≤60 seconds videos were not published until around the turn of the year 2021 – 2022. The median for reached accounts for all videos was 551 per video in 2020 – 2021 and 5,317 in 2022 – 2024. The median for views for all videos was 1,695 per video in 2020 – 2021 and 6,533 in 2022 – 2024. Imputing older posts for analysis would have underestimated the categories published since 2020. Based on these findings, the video posts published between 2022 – 2024 were included in the final analysis.


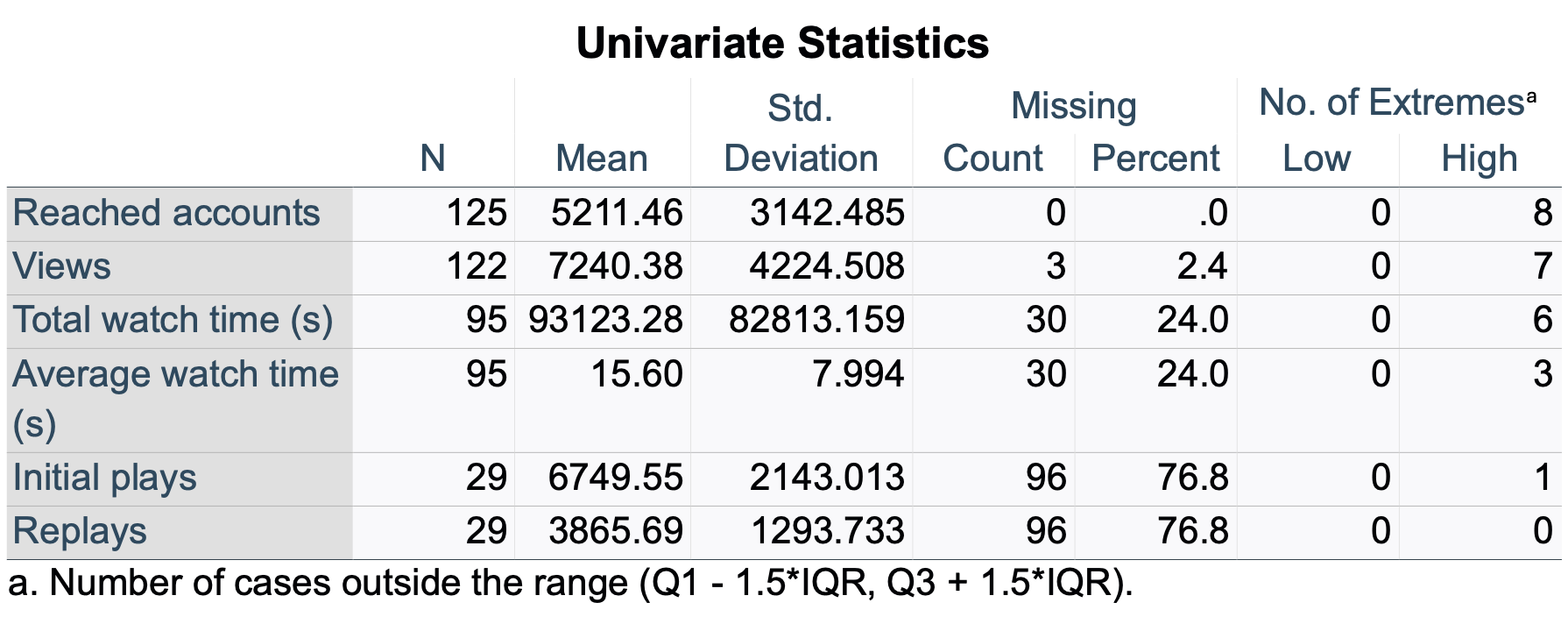


**Figure S2 | Little´s Missing Completely at Random (MCAR) test.** MCAR test was performed for all videos (n=125) included in the final analysis. Reached accounts with 0% and views with 2.4% missing data showed minor missing data. For views three missing pieces of data were recognized to cause technical reason in Instagram Insights as it did not provide views for three carousel posts (posts having more than one picture/video included to one post). For watch time and initial/replays reason for missing data were logical due to Instagram Insights development. Insights provided watch time metrics from September 2022 onwards and initial and replays from September 2023 onwards. Based on these findings, we did not replace missing data with statistical methods.

| Comparision | p-value | Ordinal | Tests | Adjusted p-value | Statistically significant |
| --- | --- | --- | --- | --- | --- |
| VIDEO LENGTH | | | | | |
| TV - CLSV | 0,542 | 6 | 6 | 0,542 | No |
| TV - HPAV | 0,001 | 1 | 6 | 0,006 | Yes |
| TV - ENT | 0,001 | 2 | 6 | 0,003 | Yes |
| CLSV - HPAV | 0,001 | 3 | 6 | 0,002 | Yes |
| CLSV - ENT | 0,001 | 4 | 6 | 0,0015 | Yes |
| HPAV - ENT | 0,03 | 5 | 6 | 0,036 | Yes |
| ANI - PHA | 0,073 | 2 | 3 | 0,1095 | No |
| ANI - HUM | 0,1 | 3 | 3 | 0,1 | No |
| PHA - HUM | 0,001 | 1 | 3 | 0,003 | Yes |
| RELATIVE WATCH TIME | | | | | |
| TV - CLSV | 0,501 | 6 | 6 | 0,501 | No |
| TV - HPAV | 0,001 | 1 | 6 | 0,006 | Yes |
| TV - ENT | 0,001 | 2 | 6 | 0,003 | Yes |
| CLSV - HPAV | 0,001 | 3 | 6 | 0,002 | Yes |
| CLSV - ENT | 0,001 | 4 | 6 | 0,0015 | Yes |
| HPAV - ENT | 0,101 | 5 | 6 | 0,1212 | No |
| ANI - PHA | 0,842 | 3 | 3 | 0,842 | No |
| ANI - HUM | 0,008 | 2 | 3 | 0,012 | Yes |
| PHA - HUM | 0,001 | 1 | 3 | 0,003 | Yes |
| >120s – 61-120s | 0,035 | 3 | 3 | 0,035 | Yes |
| >120s - ≤60s | 0,001 | 2 | 3 | 0,0015 | Yes |
| 61-120s - ≤60s | 0,001 | 1 | 3 | 0,003 | Yes |
| AVERAGE WATCH TIME | | | | | |
| >120s – 61-120s | 0,001 | 3 | 3 | 0,001 | Yes |
| >120s - ≤60s | 0,001 | 2 | 3 | 0,0015 | Yes |
| 61-120s - ≤60s | 0,001 | 1 | 3 | 0,003 | Yes |
| REACH | | | | | |
| TV - CLSV | 0,67 | 6 | 6 | 0,67 | No |
| TV - HPAV | 0,001 | 1 | 6 | 0,006 | Yes |
| TV - ENT | 0,388 | 5 | 6 | 0,4656 | No |
| CLSV - HPAV | 0,001 | 2 | 6 | 0,003 | Yes |
| CLSV - ENT | 0,1 | 3 | 6 | 0,2 | No |
| HPAV - ENT | 0,029 | 4 | 6 | 0,0435 | Yes |
| >120s – 61-120s | 0,014 | 2 | 3 | 0,021 | Yes |
| >120s - ≤60s | 0,899 | 3 | 3 | 0,899 | No |
| 61-120s - ≤60s | 0,006 | 1 | 3 | 0,018 | Yes |
| VIEWS | | | | | |
| TV - CLSV | 0,12 | 4 | 6 | 0,18 | No |
| TV - HPAV | 0,001 | 1 | 6 | 0,006 | Yes |
| TV - ENT | 0,614 | 6 | 6 | 0,614 | No |
| CLSV - HPAV | 0,001 | 2 | 6 | 0,003 | Yes |
| CLSV - ENT | 0,225 | 5 | 6 | 0,27 | No |
| HPAV - ENT | 0,064 | 3 | 6 | 0,128 | No |
| >120s – 61-120s | 0,005 | 1 | 3 | 0,015 | Yes |
| >120s - ≤60s | 0,704 | 3 | 3 | 0,704 | No |
| 61-120s - ≤60s | 0,012 | 2 | 3 | 0,018 | Yes |

**Table S3 | Adjusted p-values.** Benjamini–Hochberg post-hoc corrections were applied since the nature of the study was explorative, and we tested multiple subgroups. Post-hoc corrections were calculated in Excel version 16.103.3 (Microsoft Corporation). For p-values reported by SPSS as p < 0.001, a value of 0.001 was used for multiple-comparison correction, resulting in conservative estimates. A p-value of ≤ 0.05 was considered statistically significant. TV = theory videos, CLSV = clinical skills videos, HPAV = health promotion and awareness videos, ENV = entertainment videos, HUM = human model videos, PHA = phantom model videos and ANI = animation videos.
